# Afectación Aislada del Sexto par Craneal Como Forma de Presentación de Neuroborreliosis

**DOI:** 10.31083/RN36664

**Published:** 2025-10-28

**Authors:** Antonio José Moreno de la Bandera, Marian Vives Crook, Alejandro Ballivian Abaroa

**Affiliations:** ^1^Servicio de Neurología, Hospital Universitario Son Espases, 07120 Palma, Illes Balears, España

**Keywords:** parálisis del nervio abducens, *Borrelia burgdorferi*, eritema crónico migrans, enfermedad de Lyme, neuroborreliosis de Lyme, meningitis aséptica, abducens nerve diseases, *Borrelia burgdorferi*, erythema chronicum migrans, Lyme disease, Lyme neuroborreliosis, meningitis aseptic

## Abstract

**Introducción:**

La enfermedad de Lyme es una zoonosis causada por espiroquetas del género *Borrelia*, que presenta un amplio espectro de manifestaciones clínicas. Esto puede dificultar su diagnóstico, especialmente en regiones con baja prevalencia.

**Caso Clínico:**

Presentamos el caso de una mujer de 65 años que acudió a urgencias por diplopía de 24 horas de evolución desde el despertar, sin otra focalidad neurológica. Fue diagnosticada de neuroborreliosis tras identificar antecedentes de viaje a una zona endémica y manifestaciones cutáneas compatibles. La evolución clínica, junto con la confirmación serológica, permitió un diagnóstico precoz y la instauración de tratamiento antibiótico adecuado, con resolución completa de los síntomas.

**Conclusiones:**

Este caso resalta la necesidad de incluir la enfermedad de Lyme en el diagnóstico diferencial de pacientes con focalidad neurológica y antecedentes de viaje a áreas endémicas, incluso en ausencia de picaduras recordadas o manifestaciones clásicas. La detección precoz y el tratamiento oportuno son fundamentales para prevenir complicaciones crónicas e invalidantes.

## Abstract (en)

**Introduction:**

Lyme disease is a zoonosis caused by spirochetes of the genus *Borrelia*, presenting with a broad spectrum of clinical manifestations. This heterogeneity may hinder diagnosis, particularly in regions with low prevalence.

**Case Report:**

We present the case of a 65-year-old woman who attended the emergency department with a 24-hour history of diplopia upon awakening, without other neurological focal signs. Neuroborreliosis was diagnosed after identifying a history of travel to an endemic area and compatible cutaneous manifestations. Clinical progression, together with serological confirmation, enabled early diagnosis and initiation of appropriate antibiotic therapy, resulting in complete resolution of symptoms.

**Conclusions:**

This case highlights the importance of including Lyme disease in the differential diagnosis of patients with any neurological focal signs and a history of travel to endemic regions, even in the absence of recalled tick bites or classical manifestations. Early detection and timely treatment are crucial to prevent chronic and disabling complications.

## 1. Introducción

La enfermedad de Lyme es una zoonosis causada por espiroquetas transmitida por garrapatas del género Ixodes [1]. Consta de varias fases. En la fase inicial es típica la aparición de un cuadro pseudogripal y una dermatosis llamada eritema migrans, siendo la presentación clínica más frecuente de la enfermedad.

En España se clasifica como Enfermedad de Declaración Obligatoria. En las últimas décadas han aumentado los casos reportados y las hospitalizaciones, siendo las manifestaciones neurológicas los motivos más frecuentes de dichos ingresos [2].

La neuroborreliosis constituye la segunda presentación clínica más frecuente. Son características la radiculoneuritis, la neuritis de pares craneales y la meningitis linfocitaria.

Describimos el caso clínico de una paciente con factores de riesgo vascular con parálisis aislada del nervio que finalmente se diagnosticó de enfermedad de Lyme.

## 2. Caso Clínico

Mujer de 65 años diabética e hipertensa valorada en urgencias por diplopía no fluctuante de 24 horas de evolución desde el despertar. No había dolor en los movimientos oculares ni pérdida de agudeza visual. Además, explicaba una cefalea punzante parietal izquierda de intensidad leve. No refería otros síntomas neurológicos ni sistémicos.

**Fig. 1.**
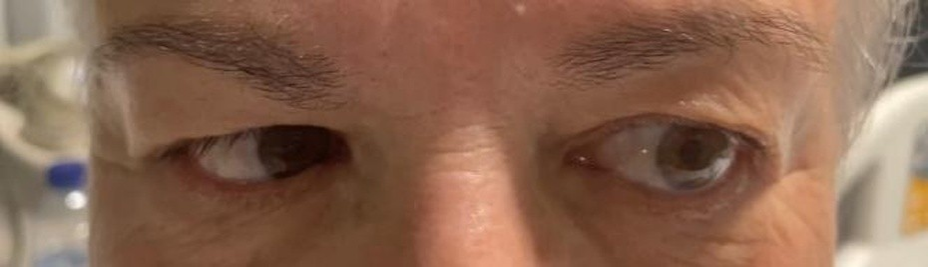
**Limitación de la abducción del ojo izquierdo (parálisis del VI par craneal)**.

A la exploración neurológica la paciente solamente presentaba una limitación en la abducción del ojo izquierdo, sugestiva de parálisis del VI par craneal (Fig. 1). No había afectación de otros pares craneales. No presentaba déficit motor ni sensitivo ni dismetrías.

Orientada inicialmente como una paresia microvascular de VI par craneal.

En la analítica de sangre destacaba una leucocitosis (11,6 × 10^9^/L) con neutrofilia del 86,73%, elevación de la proteína C reactiva (6,74 mg/dL) y procalcitonina (0,14 ng/mL). El perfil hepático y biliar estaban alterados: aspartato aminotransferasa (AST) (75 U/L), alanina aminotransferasa (ALT) (149 U/L), gamma-glutamil transferasa (GGT) (288 U/L) y fosfatasa alcalina (330 U/L) con bilirrubina total <1,2 mg/dL. Los estudios de coagulación y de función renal fueron normales.

El estudio mediante tomografía computarizada (TC) craneal sin contraste, la radiografía de tórax, el electrocardiograma, el sedimento urinario y el examen de fondo de ojo no mostraron alteraciones.

Ante estos hallazgos interrogamos de forma dirigida a la paciente: dos semanas antes había presentado un cuadro autolimitado de 4–5 días de evolución consistente en malestar general, artromialgias y fiebre de hasta 39 °C. Además, había advertido una lesión circunferencial eritematosa indolora en el brazo derecho unos días antes de la aparición de la diplopía.

**Fig. 2.**
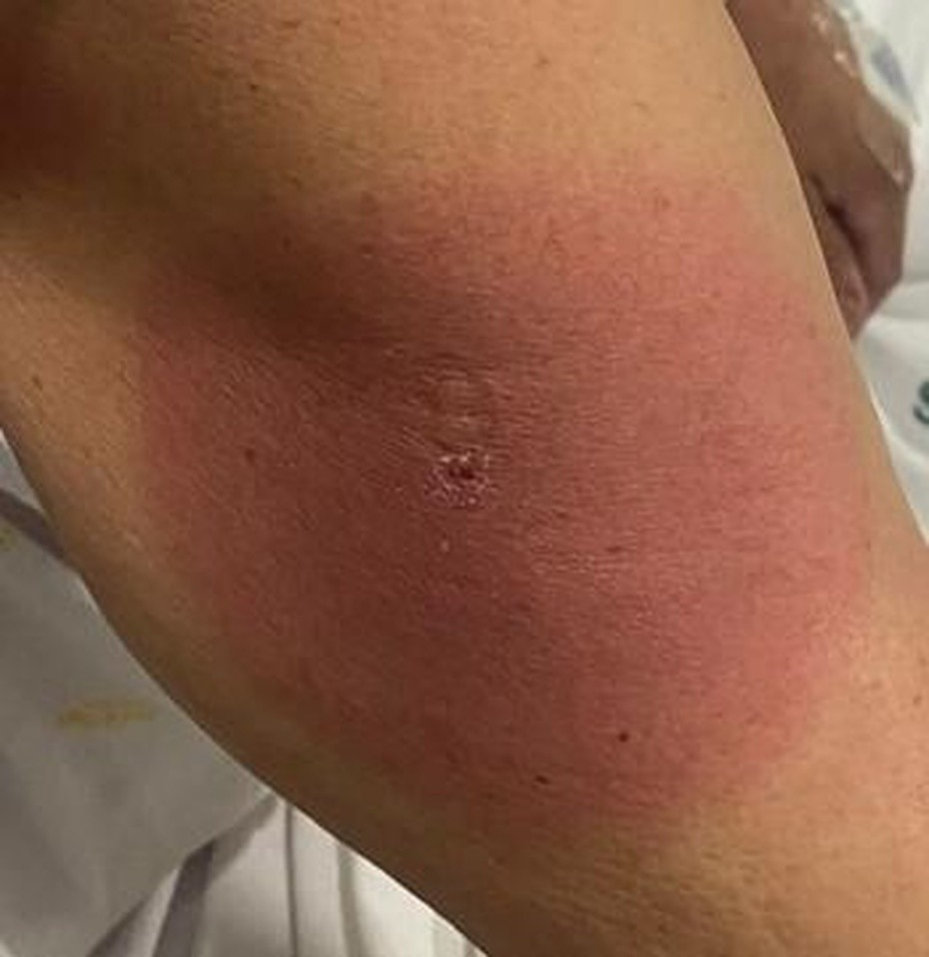
**Lesión eritematosa anular con pápula ulcerada central**.

En la exploración se objetivó una placa anular eritematosa con una costra central hemática, sugestiva de una picadura (Fig. 2).

La paciente no recordaba haber sido picada por insectos ni tener contacto con animales pero sí recordó haber estado en un entorno rural durante un viaje que hizo a Nova Scotia, Canadá hace un mes.

Debido a las lesiones cutáneas y la sintomatología neurológica se sospechó un cuadro infeccioso por lo que se solicitó serología de Borrelia, y pruebas de autoinmunidad.

La paciente presentó febrícula (37,7 °C) unas horas más tarde y extensión generalizada de lesiones eritematosas anulares por lo que se realizó una punción lumbar. El líquido cefalorraquídeo (LCR) era incoloro, con leucocitosis linfocítica (29/µL), proteínas elevadas (0,79 g/L), y glucosa algo reducida (cociente 0,56). También se observó un aumento del cociente de albúmina, aunque sin síntesis intratecal de anticuerpos IgG. Los estudios microbiológicos (Gram, cultivo, baciloscopia y reacción en cadena de la polimerasa (PCR) para virus neurotropos) fueron negativos. Sin embargo, la serología inicial (ELISA) en suero detectó positividad para IgG e IgM contra *Borrelia burgdorferi*, confirmándose posteriormente mediante técnica *Western-Blot*.

Se inició antibiótico con doxiciclina 100 mg/12 h desapareciendo las lesiones cutáneas y la diplopía en los días siguientes.

## 3. Revisión de la Literatura y Discusión

La enfermedad de Lyme es una enfermedad capaz de afectar a múltiples órganos y sistemas del cuerpo, entre ellos, el sistema nervioso.

Los estadios de la infección se dividen en fase aguda localizada, fase aguda diseminada y fase tardía. Los síntomas neurológicos aparecen fundamentalmente en la fase aguda diseminada y la tardía [3].

En el 80–85% de los pacientes aparece inicialmente una lesión cutánea llamada eritema migrans a pocos días de la picadura que remite espontáneamente en pocas semanas. Semanas o meses después, pueden aparecer manifestaciones extracutáneas dando lugar a la fase aguda diseminada [4].

La neuroborreliosis ocurre aproximadamente en el 15% de los casos [5]. En Norteamérica el cuadro más frecuente es una meningitis linfocitaria con o sin afectación de pares craneales. Inicialmente cursa con cuadro pseudogripal inespecífico dificultando el diagnóstico al confundirse con viriasis respiratorias u otras entidades benignas [6]. Aproximadamente el 60% de los casos presentan meningitis y parálisis facial [7].

En Europa es más habitual la presentación en forma de síndrome de Bannwarth que cursa con polirradiculitis dolorosa y neuritis de pares craneales; también tiende a presentar pleocitosis linfocitaria. Menos frecuentemente se produce neuropatía periférica [3].

En la literatura se describe que el par craneal más frecuentemente implicado es el VII (80–90%), que en un tercio de los casos puede ser bilateral [8]. La afectación de otros pares craneales es menos habitual, pero hay descritos casos de afectación de cualquier par craneal excepto el olfatorio (la del nervio óptico es anecdótica). Dentro de estos, los pares craneales oculomotores son los más comúnmente afectados después del nervio facial [9].

Cuando se producen neuritis de pares craneales habitualmente se acompañan de una meningitis linfocitaria y aparecen en los primeros 2 meses tras la primoinfección [7].

Pese a que nuestra paciente presentaba factores de riesgo cardiovascular y, por tanto, la parálisis del VI par podría haber sido isquémica, el hecho de que ocurriera en contexto de una meningitis linfocitaria y de la positividad de las serologías tanto para IgM como IgG hace más probable considerar que fuese debida a la infección.

Por otro lado, si bien los anticuerpos pueden persistir positivos en suero muchos años después de una infección resuelta, en nuestro caso la paciente presentaba la dermatosis característica de la enfermedad en fases iniciales que había aparecido tras el viaje reciente a una zona endémica, por lo que consideramos muy probable que la positividad de la serología fuese reciente. En la fase tardía (meses o años tras la primoinfección) las principales manifestaciones clínicas son la afectación articular y la neurológica [4].

El análisis de LCR suele mostrar un líquido claro y transparente, con discreta elevación de células de predominio linfocitario, leve elevación de proteínas y cociente de glucosa LCR/suero normal. La tinción de Gram y los cultivos bacteriológicos son negativos, por lo que frecuentemente se considera una meningitis aséptica [3].

El diagnóstico puede hacerse en aquellos pacientes que cumplan los siguientes criterios: (1) han estado en zonas endémicas; (2) presentan síntomas neurológicos típicos (meningo/radiculitis y/o neuritis de pares craneales); (3) el análisis de LCR es sugestivo de meningitis linfocitaria y (4) se obtienen dos determinaciones serológicas positivas consecutivas en suero frente a *B.burgdorferi*; la primera obtenida mediante técnica ensayo por inmunoabsorción ligado a enzimas (ELISA) y, si ésta es positiva, se confirma con una determinación mediante *Western-Blot* [3].

Si el paciente cumple todas las condiciones anteriores, como era nuestro caso, no es necesario hacer más pruebas diagnósticas [3].

En caso de duda, el diagnóstico definitivo de neuroborreliosis se realiza mediante la determinación del índice de anticuerpos que demuestre la presencia de síntesis intratecal de IgG con una relación de anticuerpos en LCR/suero de al menos 1,5. Esta prueba puede dar un resultado falso negativo si se realiza de forma precoz, ya que la síntesis de anticuerpos en LCR suele comenzar a partir de la segunda semana. La máxima sensibilidad la alcanza a partir de la semana 6, siendo de un 99% [10].

En nuestro caso no se solicitó específicamente síntesis de anticuerpos anti-*B.burgdorferi*, si bien el índice de Tibbling link no reveló síntesis intratecal general de IgG.

El tratamiento de elección de la neuroborreliosis es la doxiciclina: 100 mg/12 h por vía oral durante 14 días. La doxiciclina oral ha demostrado ser igual de efectiva para el tratamiento de neuroborreliosis que la ceftriaxona endovenosa. La mayoría de los pacientes responden muy favorablemente al tratamiento. Otra alternativa antibiótica es la amoxicilina [11].

Nuestro caso tiene la peculiaridad de que la paciente presentaba una afectación selectiva de sexto par craneal, sin afectación del nervio facial, radiculitis ni otros signos característicos de la enfermedad.

## 4. Conclusiones

La enfermedad de Lyme es un reto diagnóstico debido a que la clínica de la fase inicial puede pasar desapercibida y el rango de manifestaciones neurológicas es muy variable. Sin un tratamiento adecuado, se puede cronificar y volverse invalidante.

Este caso subraya la necesidad de considerar la enfermedad de Lyme en pacientes con antecedentes de viajes a áreas endémicas y cualquier focalidad neurológica posterior, incluso si no presentan síntomas clásicos o no recuerdan picaduras de insectos. La evolución clínica del paciente, que incluye la aparición de una lesión eritematosa y síntomas neurológicos, sugirió una neuroborreliosis. El reconocimiento temprano de los signos clínicos y la historia de viaje son esenciales para un diagnóstico preciso y una intervención adecuada.

## Data Availability

Los datos que respaldan los hallazgos de este estudio están disponibles a pedido razonable del autor correspondiente.

## Contribuciones de los Autores

ABA diseñó el estudio. AJMdlB y MVC recogieron los datos clínicos. AJMdlB hizo la revisión bibliográfica y la primera redacción del manuscrito. MVC y ABA analizaron críticamente los datos. Todos los autores contribuyeron a los cambios editoriales del manuscrito. Todos leyeron y aprobaron el manuscrito final. Todos los autores participaron lo suficiente en el trabajo y aceptaron ser responsables de todos sus aspectos.

## Aprobación Ética y Consentimiento Informado

Se obtuvo el consentimiento informado por escrito de la paciente antes de publicar este caso clínico y las imágenes que lo acompañan. El estudio se ha llevado a cabo conforme a la Declaración de Helsinki y fue aprobado por el Comité de Ética de la Investigación de las Islas Baleares, con número IB 5707/25.

## Agradecimientos

No aplicable.

## Financiación

Esta investigación no recibió financiación externa.
